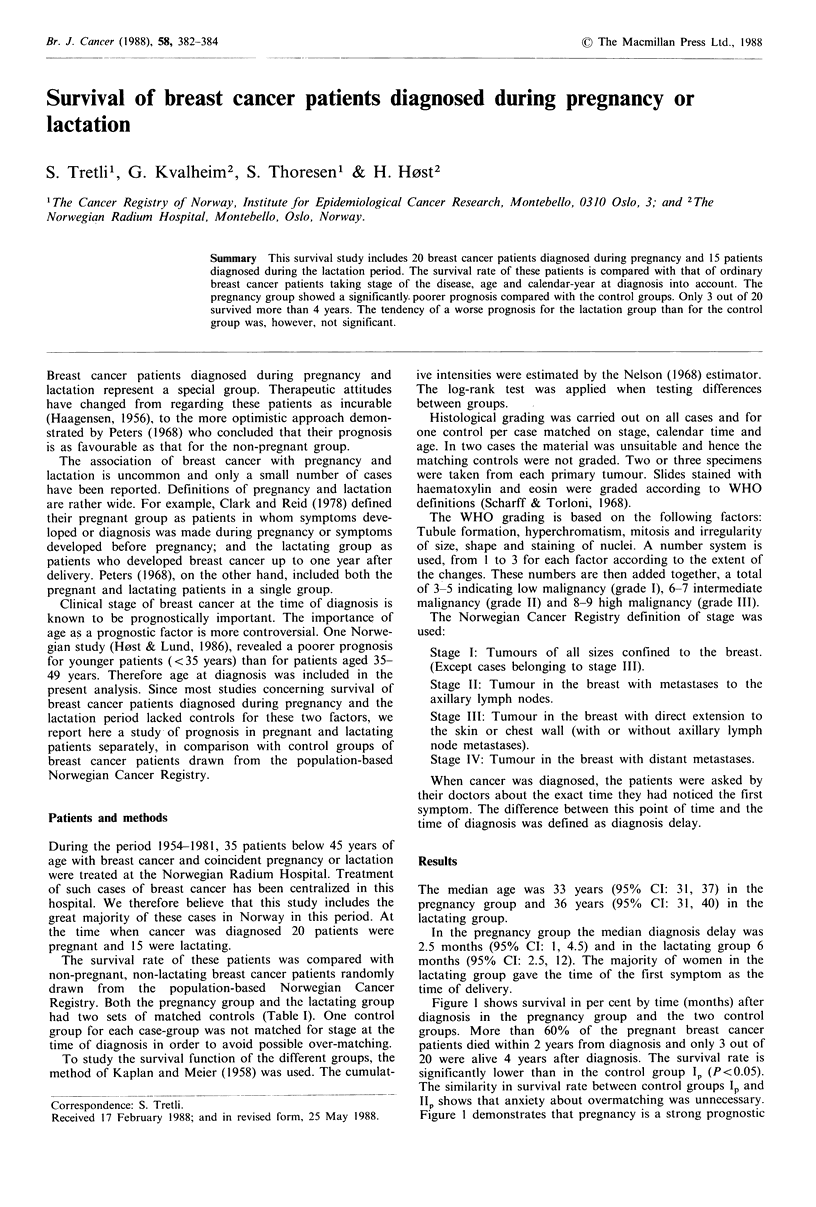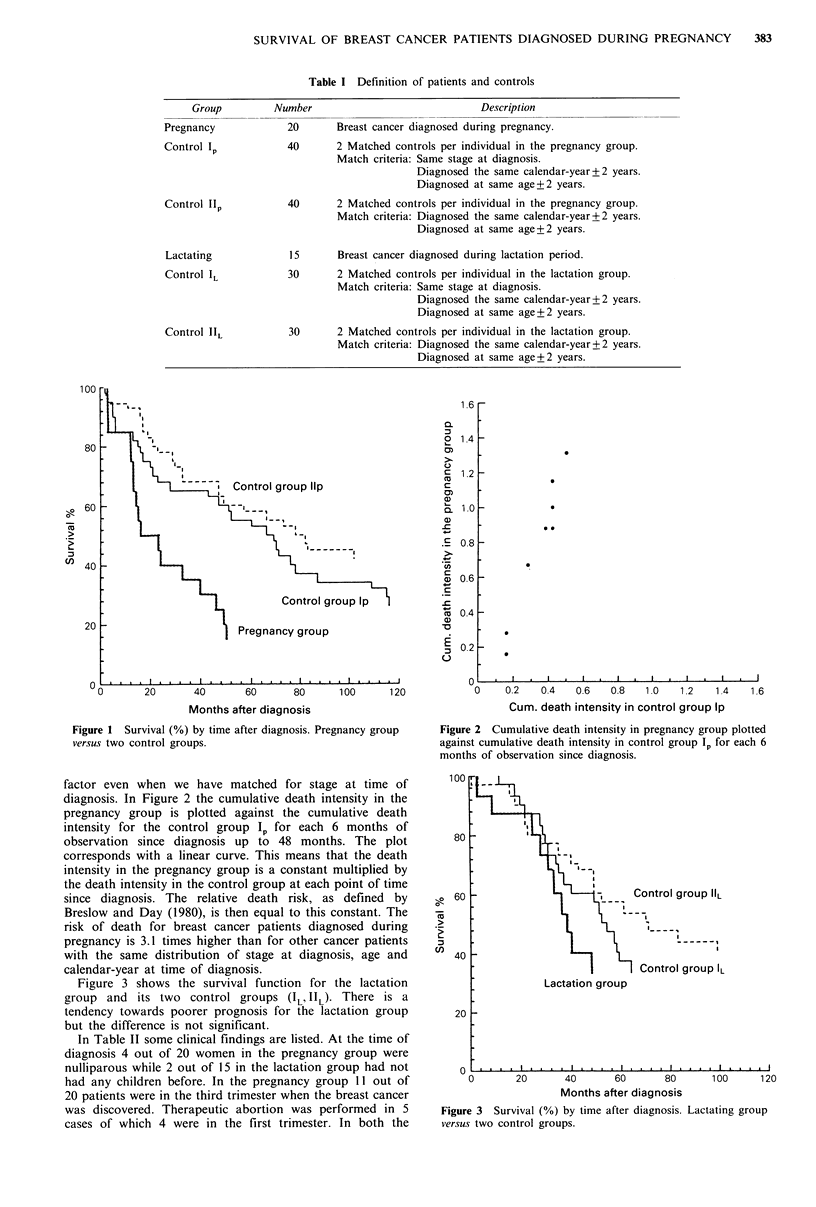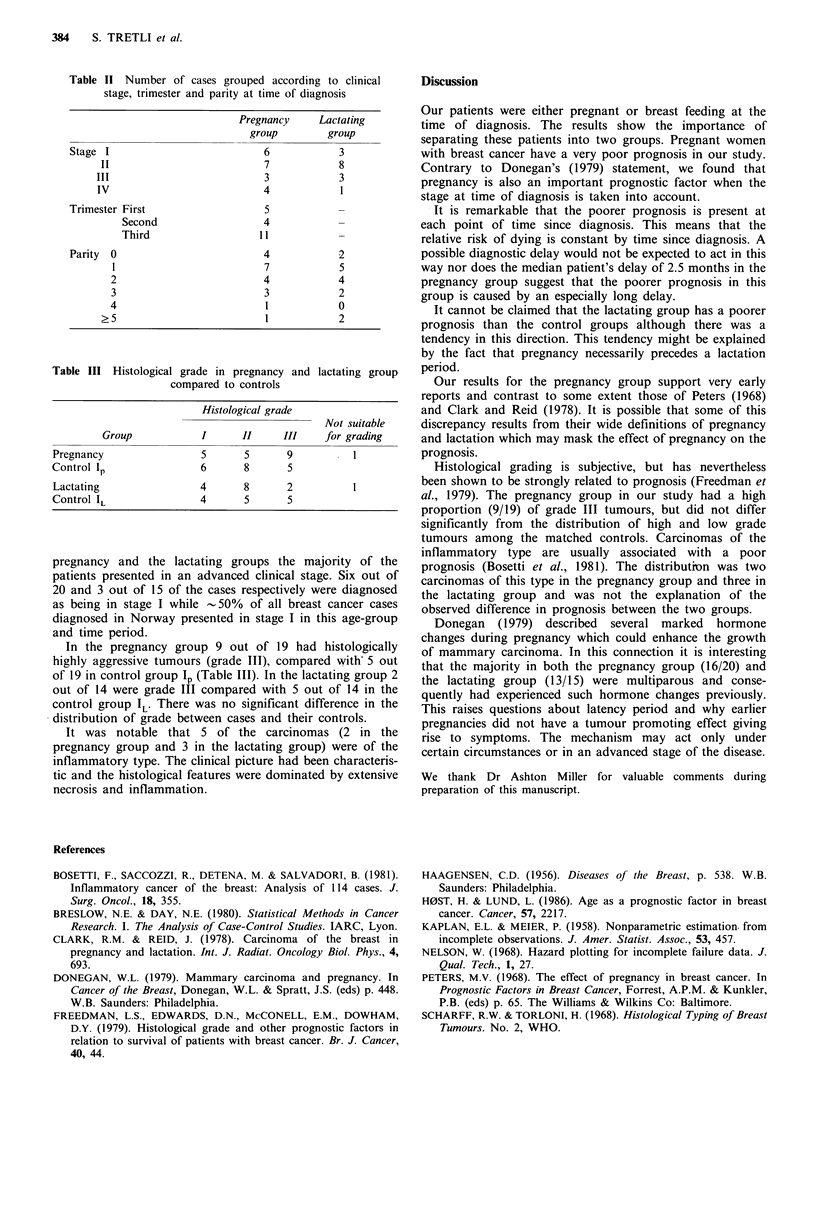# Survival of breast cancer patients diagnosed during pregnancy or lactation.

**DOI:** 10.1038/bjc.1988.224

**Published:** 1988-09

**Authors:** S. Tretli, G. Kvalheim, S. Thoresen, H. HÃ¸st

**Affiliations:** Cancer Registry of Norway, Institute for Epidemiological Cancer Research, Oslo.

## Abstract

This survival study includes 20 breast cancer patients diagnosed during pregnancy and 15 patients diagnosed during the lactation period. The survival rate of these patients is compared with that of ordinary breast cancer patients taking stage of the disease, age and calendar-year at diagnosis into account. The pregnancy group showed a significantly poorer prognosis compared with the control groups. Only 3 out of 20 survived more than 4 years. The tendency of a worse prognosis for the lactation group than for the control group was, however, not significant.


© The Macmillan Press Ltd., 1988

### Survival of breast cancer patients diagnosed during pregnancy or lactation

S. Tretli[1], G. Kvalheim[2], S. Thoresen[1] & H. Høst[2]

[1]*The Cancer Registry of Norway, Institute for Epidemiological Cancer Research, Montebello, 0310 Oslo, 3; and* [2]*The Norwegian Radium Hospital, Montebello, Oslo, Norway.*

**Summary** This survival study includes 20 breast cancer patients diagnosed during pregnancy and 15 patients diagnosed during the lactation period. The survival rate of these patients is compared with that of ordinary breast cancer patients taking stage of the disease, age and calendar-year at diagnosis into account. The pregnancy group showed a significantly poorer prognosis compared with the control groups. Only 3 out of 20 survived more than 4 years. The tendency of a worse prognosis for the lactation group than for the control group was, however, not significant.

Breast cancer patients diagnosed during pregnancy and lactation represent a special group. Therapeutic attitudes have changed from regarding these patients as incurable (Haagensen, 1956), to the more optimistic approach demonstrated by Peters (1968) who concluded that their prognosis is as favourable as that for the non-pregnant group.

The association of breast cancer with pregnancy and lactation is uncommon and only a small number of cases have been reported. Definitions of pregnancy and lactation are rather wide. For example, Clark and Reid (1978) defined their pregnant group as patients in whom symptoms developed or diagnosis was made during pregnancy or symptoms developed before pregnancy; and the lactating group as patients who developed breast cancer up to one year after delivery. Peters (1968), on the other hand, included both the pregnant and lactating patients in a single group.

Clinical stage of breast cancer at the time of diagnosis is known to be prognostically important. The importance of age as a prognostic factor is more controversial. One Norwegian study (Høst & Lund, 1986), revealed a poorer prognosis for younger patients (<35 years) than for patients aged 35–49 years. Therefore age at diagnosis was included in the present analysis. Since most studies concerning survival of breast cancer patients diagnosed during pregnancy and the lactation period lacked controls for these two factors, we report here a study of prognosis in pregnant and lactating patients separately, in comparison with control groups of breast cancer patients drawn from the population-based Norwegian Cancer Registry.

### Patients and methods

During the period 1954–1981, 35 patients below 45 years of age with breast cancer and coincident pregnancy or lactation were treated at the Norwegian Radium Hospital. Treatment of such cases of breast cancer has been centralized in this hospital. We therefore believe that this study includes the great majority of these cases in Norway in this period. At the time when cancer was diagnosed 20 patients were pregnant and 15 were lactating.

The survival rate of these patients was compared with non-pregnant, non-lactating breast cancer patients randomly drawn from the population-based Norwegian Cancer Registry. Both the pregnancy group and the lactating group had two sets of matched controls (Table I). One control group for each case-group was not matched for stage at the time of diagnosis in order to avoid possible over-matching.

To study the survival function of the different groups, the method of Kaplan and Meier (1958) was used. The cumulative intensities were estimated by the Nelson (1968) estimator. The log-rank test was applied when testing differences between groups.

Histological grading was carried out on all cases and for one control per case matched on stage, calendar time and age. In two cases the material was unsuitable and hence the matching controls were not graded. Two or three specimens were taken from each primary tumour. Slides stained with haematoxylin and eosin were graded according to WHO definitions (Scharff & Torloni, 1968).

The WHO grading is based on the following factors: Tubule formation, hyperchromatism, mitosis and irregularity of size, shape and staining of nuclei. A number system is used, from 1 to 3 for each factor according to the extent of the changes. These numbers are then added together, a total of 3–5 indicating low malignancy (grade I), 6–7 intermediate malignancy (grade II) and 8–9 high malignancy (grade III).

The Norwegian Cancer Registry definition of stage was used:

Stage I: Tumours of all sizes confined to the breast. (Except cases belonging to stage III).

Stage II: Tumour in the breast with metastases to the axillary lymph nodes.

Stage III: Tumour in the breast with direct extension to the skin or chest wall (with or without axillary lymph node metastases).

Stage IV: Tumour in the breast with distant metastases.

When cancer was diagnosed, the patients were asked by their doctors about the exact time they had noticed the first symptom. The difference between this point of time and the time of diagnosis was defined as diagnosis delay.

### Results

The median age was 33 years (95% CI: 31, 37) in the pregnancy group and 36 years (95% CI: 31, 40) in the lactating group.

In the pregnancy group the median diagnosis delay was 2.5 months (95% CI: 1, 4.5) and in the lactating group 6 months (95% CI: 2.5, 12). The majority of women in the lactating group gave the time of the first symptom as the time of delivery.

Figure 1 shows survival in per cent by time (months) after diagnosis in the pregnancy group and the two control groups. More than 60% of the pregnant breast cancer patients died within 2 years from diagnosis and only 3 out of 20 were alive 4 years after diagnosis. The survival rate is significantly lower than in the control group $I_p$ ($P<0.05$). The similarity in survival rate between control groups $I_p$ and $II_p$ shows that anxiety about overmatching was unnecessary. Figure 1 demonstrates that pregnancy is a strong prognostic

Correspondence: S. Tretli.
Received 17 February 1988; and in revised form, 25 May 1988.

**Table I** Definition of patients and controls

| *Group* | *Number* | *Description* |
|---|---|---|
| Pregnancy | 20 | Breast cancer diagnosed during pregnancy. |
| Control $I_p$ | 40 | 2 Matched controls per individual in the pregnancy group.<br>Match criteria: Same stage at diagnosis.<br>Diagnosed the same calendar-year ± 2 years.<br>Diagnosed at same age ± 2 years. |
| Control $II_p$ | 40 | 2 Matched controls per individual in the pregnancy group.<br>Match criteria: Diagnosed the same calendar-year ± 2 years.<br>Diagnosed at same age ± 2 years. |
| Lactating | 15 | Breast cancer diagnosed during lactation period. |
| Control $I_L$ | 30 | 2 Matched controls per individual in the lactation group.<br>Match criteria: Same stage at diagnosis.<br>Diagnosed the same calendar-year ± 2 years.<br>Diagnosed at same age ± 2 years. |
| Control $II_L$ | 30 | 2 Matched controls per individual in the lactation group.<br>Match criteria: Diagnosed the same calendar-year ± 2 years.<br>Diagnosed at same age ± 2 years. |

**Figure 1** Survival (%) by time after diagnosis. Pregnancy group *versus* two control groups.

**Figure 2** Cumulative death intensity in pregnancy group plotted against cumulative death intensity in control group $I_p$ for each 6 months of observation since diagnosis.

factor even when we have matched for stage at time of diagnosis. In Figure 2 the cumulative death intensity in the pregnancy group is plotted against the cumulative death intensity for the control group $I_p$ for each 6 months of observation since diagnosis up to 48 months. The plot corresponds with a linear curve. This means that the death intensity in the pregnancy group is a constant multiplied by the death intensity in the control group at each point of time since diagnosis. The relative death risk, as defined by Breslow and Day (1980), is then equal to this constant. The risk of death for breast cancer patients diagnosed during pregnancy is 3.1 times higher than for other cancer patients with the same distribution of stage at diagnosis, age and calendar-year at time of diagnosis.

Figure 3 shows the survival function for the lactation group and its two control groups ($I_L$, $II_L$). There is a tendency towards poorer prognosis for the lactation group but the difference is not significant.

In Table II some clinical findings are listed. At the time of diagnosis 4 out of 20 women in the pregnancy group were nulliparous while 2 out of 15 in the lactation group had not had any children before. In the pregnancy group 11 out of 20 patients were in the third trimester when the breast cancer was discovered. Therapeutic abortion was performed in 5 cases of which 4 were in the first trimester. In both the

**Figure 3** Survival (%) by time after diagnosis. Lactating group *versus* two control groups.

**Table II** Number of cases grouped according to clinical stage, trimester and parity at time of diagnosis

| | | *Pregnancy group* | *Lactating group* |
|---|---|---|---|
| Stage | I | 6 | 3 |
| | II | 7 | 8 |
| | III | 3 | 3 |
| | IV | 4 | 1 |
| Trimester | First | 5 | – |
| | Second | 4 | – |
| | Third | 11 | – |
| Parity | 0 | 4 | 2 |
| | 1 | 7 | 5 |
| | 2 | 4 | 4 |
| | 3 | 3 | 2 |
| | 4 | 1 | 0 |
| | ≥5 | 1 | 2 |

**Table III** Histological grade in pregnancy and lactating group compared to controls

| *Group* | *Histological grade* *I* | *II* | *III* | *Not suitable for grading* |
|---|---|---|---|---|
| Pregnancy | 5 | 5 | 9 | 1 |
| Control $I_p$ | 6 | 8 | 5 | |
| Lactating | 4 | 8 | 2 | 1 |
| Control $I_L$ | 4 | 5 | 5 | |

pregnancy and the lactating groups the majority of the patients presented in an advanced clinical stage. Six out of 20 and 3 out of 15 of the cases respectively were diagnosed as being in stage I while ~50% of all breast cancer cases diagnosed in Norway presented in stage I in this age-group and time period.

In the pregnancy group 9 out of 19 had histologically highly aggressive tumours (grade III), compared with 5 out of 19 in control group $I_p$ (Table III). In the lactating group 2 out of 14 were grade III compared with 5 out of 14 in the control group $I_L$. There was no significant difference in the distribution of grade between cases and their controls.

It was notable that 5 of the carcinomas (2 in the pregnancy group and 3 in the lactating group) were of the inflammatory type. The clinical picture had been characteristic and the histological features were dominated by extensive necrosis and inflammation.

### Discussion

Our patients were either pregnant or breast feeding at the time of diagnosis. The results show the importance of separating these patients into two groups. Pregnant women with breast cancer have a very poor prognosis in our study. Contrary to Donegan's (1979) statement, we found that pregnancy is also an important prognostic factor when the stage at time of diagnosis is taken into account.

It is remarkable that the poorer prognosis is present at each point of time since diagnosis. This means that the relative risk of dying is constant by time since diagnosis. A possible diagnostic delay would not be expected to act in this way nor does the median patient's delay of 2.5 months in the pregnancy group suggest that the poorer prognosis in this group is caused by an especially long delay.

It cannot be claimed that the lactating group has a poorer prognosis than the control groups although there was a tendency in this direction. This tendency might be explained by the fact that pregnancy necessarily precedes a lactation period.

Our results for the pregnancy group support very early reports and contrast to some extent those of Peters (1968) and Clark and Reid (1978). It is possible that some of this discrepancy results from their wide definitions of pregnancy and lactation which may mask the effect of pregnancy on the prognosis.

Histological grading is subjective, but has nevertheless been shown to be strongly related to prognosis (Freedman *et al.*, 1979). The pregnancy group in our study had a high proportion (9/19) of grade III tumours, but did not differ significantly from the distribution of high and low grade tumours among the matched controls. Carcinomas of the inflammatory type are usually associated with a poor prognosis (Bosetti *et al.*, 1981). The distribution was two carcinomas of this type in the pregnancy group and three in the lactating group and was not the explanation of the observed difference in prognosis between the two groups.

Donegan (1979) described several marked hormone changes during pregnancy which could enhance the growth of mammary carcinoma. In this connection it is interesting that the majority in both the pregnancy group (16/20) and the lactating group (13/15) were multiparous and consequently had experienced such hormone changes previously. This raises questions about latency period and why earlier pregnancies did not have a tumour promoting effect giving rise to symptoms. The mechanism may act only under certain circumstances or in an advanced stage of the disease.

We thank Dr Ashton Miller for valuable comments during preparation of this manuscript.

### References

BOSETTI, F., SACCOZZI, R., DETENA, M. & SALVADORI, B. (1981). Inflammatory cancer of the breast: Analysis of 114 cases. *J. Surg. Oncol.*, **18,** 355.

BRESLOW, N.E. & DAY, N.E. (1980). *Statistical Methods in Cancer Research. I. The Analysis of Case-Control Studies.* IARC, Lyon.

CLARK, R.M. & REID, J. (1978). Carcinoma of the breast in pregnancy and lactation. *Int. J. Radiat. Oncology Biol. Phys.*, **4,** 693.

DONEGAN, W.L. (1979). Mammary carcinoma and pregnancy. In *Cancer of the Breast*, Donegan, W.L. & Spratt, J.S. (eds) p. 448. W.B. Saunders: Philadelphia.

FREEDMAN, L.S., EDWARDS, D.N., McCONELL, E.M., DOWHAM, D.Y. (1979). Histological grade and other prognostic factors in relation to survival of patients with breast cancer. *Br. J. Cancer*, **40,** 44.

HAAGENSEN, C.D. (1956). *Diseases of the Breast*, p. 538. W.B. Saunders: Philadelphia.

HØST, H. & LUND, L. (1986). Age as a prognostic factor in breast cancer. *Cancer*, **57,** 2217.

KAPLAN, E.L. & MEIER, P. (1958). Nonparametric estimation from incomplete observations. *J. Amer. Statist. Assoc.*, **53,** 457.

NELSON, W. (1968). Hazard plotting for incomplete failure data. *J. Qual. Tech.*, **1,** 27.

PETERS, M.V. (1968). The effect of pregnancy in breast cancer. In *Prognostic Factors in Breast Cancer*, Forrest, A.P.M. & Kunkler, P.B. (eds) p. 65. The Williams & Wilkins Co: Baltimore.

SCHARFF, R.W. & TORLONI, H. (1968). *Histological Typing of Breast Tumours.* No. 2, WHO.